# Assessing the Implications of Deforestation and Climate Change on Rural Livelihood in Ghana: a Multidimensional Analysis and Solution-Based Approach

**DOI:** 10.1007/s00267-024-02053-6

**Published:** 2024-09-27

**Authors:** Richard Kwame Adom, Memory Reid, Gbenga Abayomi Afuye, Mulala Danny Simatele

**Affiliations:** 1 School of Geography, Archaeology and Environmental Studies, University of Witwatersrand, Johannesburg, South Africa; 2 The Global Change Institute (GCI), University of Witwatersrand, Johannesburg, South Africa; 3 Department of Geography and Environmental Science, University of Fort Hare, Alice, Eastern Cape Province South Africa; 4 Geospatial Application, Climate Change and Environmental Sustainability Lab–GACCES, University of Fort Hare, Alice, Eastern Cape Province South Africa

**Keywords:** Deforestation, Climate change adaptation, Livelihoods, Sustainability, Ghana

## Abstract

The Ashanti region in Ghana, abundant in natural resources such as forests and vegetation biomes, significantly supports the livelihoods of a significant portion of the population. The sustainable management of forest resources remains a significant challenge to achieving environmental and economic growth and poverty alleviation. The study aims to identify the drivers of deforestation and assess its impact on the livelihoods of the poor and vulnerable communities in the Ashanti region. The study utilized qualitative and space-based data to examine the patterns of vegetation cover and deforestation from 2000 to 2020. The results revealed moderate to sparse vegetation in Ashanti from 2002, 2005, 2011, 2015, 2017, and 2018, with no vegetation in the northcentral part, attributed to climate change, agricultural practices, government policies, and deforestation-related disasters. The study found a significant correlation (*R*² = 0.8197) between years and deforestation areas, especially in 2018 at around 16,000 Sqkm, indicating an exponential increase with severe implications for sustainable livelihoods. Much of these changes were reflected in 2020 with a high peak of deforestation towards the southeastern parts of the region. Additionally, the results show that the poor groups are not passive actors but are actively involved in identifying systems and processes through which to build their adaptive capacity and resilience to environmental and climate change-induced changes. The findings provide evidence-based and all-inclusive approaches that would encourage vulnerable and marginalized groups to participate in the co-production and co-creation of policies and strategies. This outcome is geared towards transformative and sustainable communities while ensuring efficient and effective response and recovery capacities of deforested lands.

## Introduction

One of the greatest environmental challenges facing the world today is deforestation (Bologna, Aquiro (2020)). Woodlands and forests play a crucial role in Earth’s climate resilience, acting as carbon sinks, slowing global warming, contributing to the hydrological cycle, and providing atmospheric moisture through transpiration (Lawrence et al. 2022). Similarly, forests act as natural water filters and regulators, maintaining freshwater resources, reducing soil erosion, sifting pollutants, and regulating water flow (Lawrence et al. 2022; Shah et al. 2022). Forests and forest products provide livelihoods for over a billion people globally, including timber, non-timber products, and fuelwood, particularly in developing nations (Zhu et al. 2023). Forests offer crucial ecosystem services, supporting agricultural production, water generation, soil fertility, and non-timber forest product provision. Forests and agriculture are crucial for the economic development and livelihood support of people in various countries. Accordingly, forest products and activities generate $250 billion annually globally, supporting numerous local, national, and international economies through tourism, trade, and manufacturing (Arce 2019). Therefore, forests significantly contribute to global income generation and household food security (Amoah and Korle 2020).

Despite the numerous benefits associated with forests and woodlands, the rate at which forests are disappearing across the globe is alarming (Ritchie and Roser 2024). Many scholars describe deforestation as the process of clearing forests for various purposes such as agriculture, settlement, infrastructure development, and mining (Lund 2018; de Oca et al. 2021; Brandt et al. 2022). Boafo (2013) also defined deforestation as the conversion of forested land for other purposes or a permanent reduction in canopy cover. Global deforestation, primarily in developing nations, causes 13 million hectares of land loss annually, accounting for 31% of the world’s total forest cover, with countries like Brazil, Indonesia, Congo, Cameroon, Australia, the US, and Bolivia contributing over 60% (Boafo 2013; Brandt et al. 2022; Lawrence et al. 2022). Ritchie (2021) reported a 4.7 million hectare annual net loss of global forest since 2010, a figure comparable to the combined size of the United States and China. Many researchers and environmental advocators attributed deforestation to various factors, including farming practices, land use, development, and natural factors like weather extremes, according to researchers and environmental advocates (Prevedello et al. 2019; Pickering, Guglyuvatyy (2019); Cadman et al. 2019). Igini (2022) identified poor farming techniques, mining, infrastructure development, and urban expansion as the primary contributors to global deforestation. Global deforestation causes biodiversity loss, climate uncertainty, water cycle disruption, soil erosion, Indigenous community displacement, land rights disputes, and health impacts due to cultural and physical displacement (Butler 2019; Eiras-Barca et al. (2020)). Deforestation leads to short-term economic benefits but long-term costs, including reduced agricultural outputs, increased unemployment, and decreased revenue in ecotourism, trade, and industries (Arce 2019).

Ghana covers 35% of the total land area, with 7.9 million hectares of forested land (Kyere-Boateng and Marek 2021). In 2022, Ghana experienced a 70% increase in the loss of 18,000 hectares of its primary forest, marking the largest loss in recent years (Afele et al. 2021). Boafo (2013) attributed 95% of deforestation in Ghana to demographic and economic pressures, policy, institutional lapses, increased land demand, fuel wood, illegal logging, mining, infrastructure development, and community expansion. The Food and Agriculture Organization of the United Nations (FAO) reports that 96.25% of 7.6 million hectares are primary or naturally regenerated forests, while 3.75% of 297,000 hectares are planted forests (FAO 2020; Afele et al. 2021). Boafo (2013) and Kyere-Boateng et al. (2021) identified cash crop cultivation, illegal lumbering, mining operations, and settlement expansion as primary causes of deforestation in Nigeria’s Southern regions, alongside unsustainable charcoal production and forest fires. Acheampong et al. (2019) and Sasu (2022) report that over 60% of Ghana’s primary forests have been lost, with an annual deforestation rate of 3.51%, resulting in over 315,000 hectares of loss and projected to surpass 4.7 million by 2030. Natural factors like hurricanes, fires, parasites, and floods contribute to deforestation across the country (Amoah and Korle 2020).

Deforestation in the country has severe environmental and human well-being impacts, affecting sustainable livelihoods, disrupting environmental functions, and destroying forest ecosystems (Afele et al. 2022). Deforestation in Ghana poses significant socioeconomic risks due to climate unpredictability, soil degradation, species extinction, and rapid decline in the river and stream water levels (Khodadadi et al. 2021). Kyere-Boateng et al. (2021) posits that between 1990 and 2021, over 200 deforestation-related disasters occurred, resulting in over 100,000 deaths and $2 billion in economic losses, equivalent to 0.5% of Ghana’s GDP. Chirwa and Adeyemi (2020) found that the agriculture and forestry sectors employ over 60% of the population, including 53% of women, resulting in an indirect cost of over US$400 million, equivalent to 0.7% of 2020 GDP. Previous studies have explored the social and economic impacts of deforestation on livelihoods, the need for policy reforms, socioeconomic issues, governance, and advancements in deforestation rate analysis, among other topics (Boafo, 2013; Khodadadi et al. 2021; Adom et al. 2023). Amoah and Korle (2020) and Acheampong et al. (2019) conducted studies on deforestation in Ghana, using remote sensing techniques, and suggested modifications to farming techniques to increase yield. Numerous studies have extensively explored socioeconomic factors linked to deforestation, governance, and advancements in deforestation rate analysis techniques (Boafo 2013; Acheampong et al. 2019; Amoah and Korle 2020; Afele et al. 2022). Nevertheless, there is a lack of literature on community-based forest management strategies and adaptations for the long-term social and economic impacts of deforestation on Ghana’s economic development. Hence, this study explores the impact of deforestation and climate change on the socioeconomic livelihoods of the poor and rural population in Ghana, filling a gap in the existing literature.

## Theoretical Perspectives on Drivers of Deforestation and its Impacts on Climate Change in Ghana

**Fig. 1:**
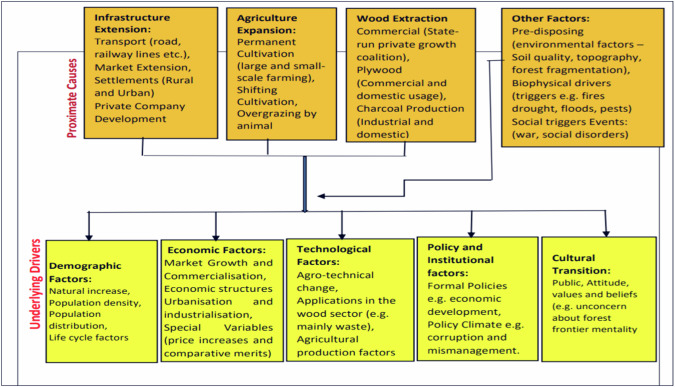
The underlying causes of deforestation in Ghana. Source: Caroline Sartorato Silva França

Figure 1 provides a detailed explanation of the root and proximate causes of deforestation, specifically in Ghana. The factors driving the proximate causes of forest degradation can be categorized into six areas: demographic factors, economic factors, technological factors, policy and institutional factors, and cultural factors. Demographic factors include natural increases in fertility and mortality rates, migration patterns, population density, distribution, and life cycle features. Economic factors include market growth, commercialization, urbanization, and special variables like price increases and comparative cost advantages. Technological factors include agro-technical change, technological advancements in the wood sector, and changes in agricultural inputs and methods. Policy and institutional factors include formal policies, policy climate, property rights, public attitudes, values, and beliefs, and individual and household behavior driven by concern or rent-seeking behaviors. Tacconi et al. (2019) highlight illegal practices in the forest sector, including government officers authorizing contracts, illegal harvesting, under-declaring forest product volumes, underpricing of timber, and land invasion. The increase in illegal forest practices has been detrimental to the conservation of forests. Therefore, Understanding these causes can aid in developing comprehensive strategies to combat deforestation.

Illegal forest practices, such as government-authorized contracts, illegal harvesting license sales, under-declaration of forest product volumes, under-pricing of timber, and invasion of protected land, significantly impact forest conservation (Tacconi et al. 2019). Moreover, deforestation in Ghana is primarily driven by poverty, economic factors like logging, expanding land for food production, fuel wood extraction, and both legal and illegal mining operations (Gyamfi et al. 2021). Figure 1 shows the complex interplay of immediate activities and deep-rooted systemic factors that contribute to deforestation in Ghana. Accordingly, Ghana’s licensed timber production companies are legally authorized to harvest timber in compliance with government regulations (Astana et al. 2020). The Forestry Commission of Ghana mandates that these companies must adhere to sustainable practices and guidelines (Frey et al. 2021). The rise in production activities of these companies has led to an increase in the Annual Allowable Cut (AAC) (Astana et al. 2020; Frey et al. 2021). However, the maximum volume of timber legally harvested within a year is monitored to ensure sustainable forest management. Legally licensed companies have increased production in recent years to meet domestic and international timber demand and control illegal logging. The balance between increased AAC and forest resources must be managed effectively to prevent overexploitation and degradation. On the other hand, non-compliance with Ghana’s regulations could lead to severe environmental and economic consequences, including overexploitation of forest resources, deforestation, biodiversity loss, ecosystem disruption, reduced carbon sequestration, and worsened climate change. Sustainable practices are crucial for maintaining ecological balance, economic stability, and social well-being, but communities reliant on forests may face threats.

Scholars like Afele et al. (2021), Fagariba et al. (2018), and Teye (2008), argued that deforestation in Ghana is influenced by five main factors rather than one single variable. Human activities at the local or community level, such as agricultural expansion, illegal mining, logging, and forest products, are primarily driven by increasing food production and population growth (Peprah et al. 2022). Deforestation in Ghana is influenced by social, demographic, economic, technological, policy institutional, and cultural factors, creating a complex system that drives deforestation activities, as postulated by Nyako et al. (2023) and Asamoah et al. (2023). Fagariba et al. (2018) argue that rural poverty and population growth lead to the expansion of agriculture and illicit mining, often exacerbated by weak land tenure policies, inadequate enforcement, and cultural practices that shape individual and community behavior towards forests. Figure 1 summarizes the key factors that drive deforestation in Ghana.

### The Implication of Deforestation on the Environment and Sustainable Livelihoods in Ghana

**Fig. 2:**
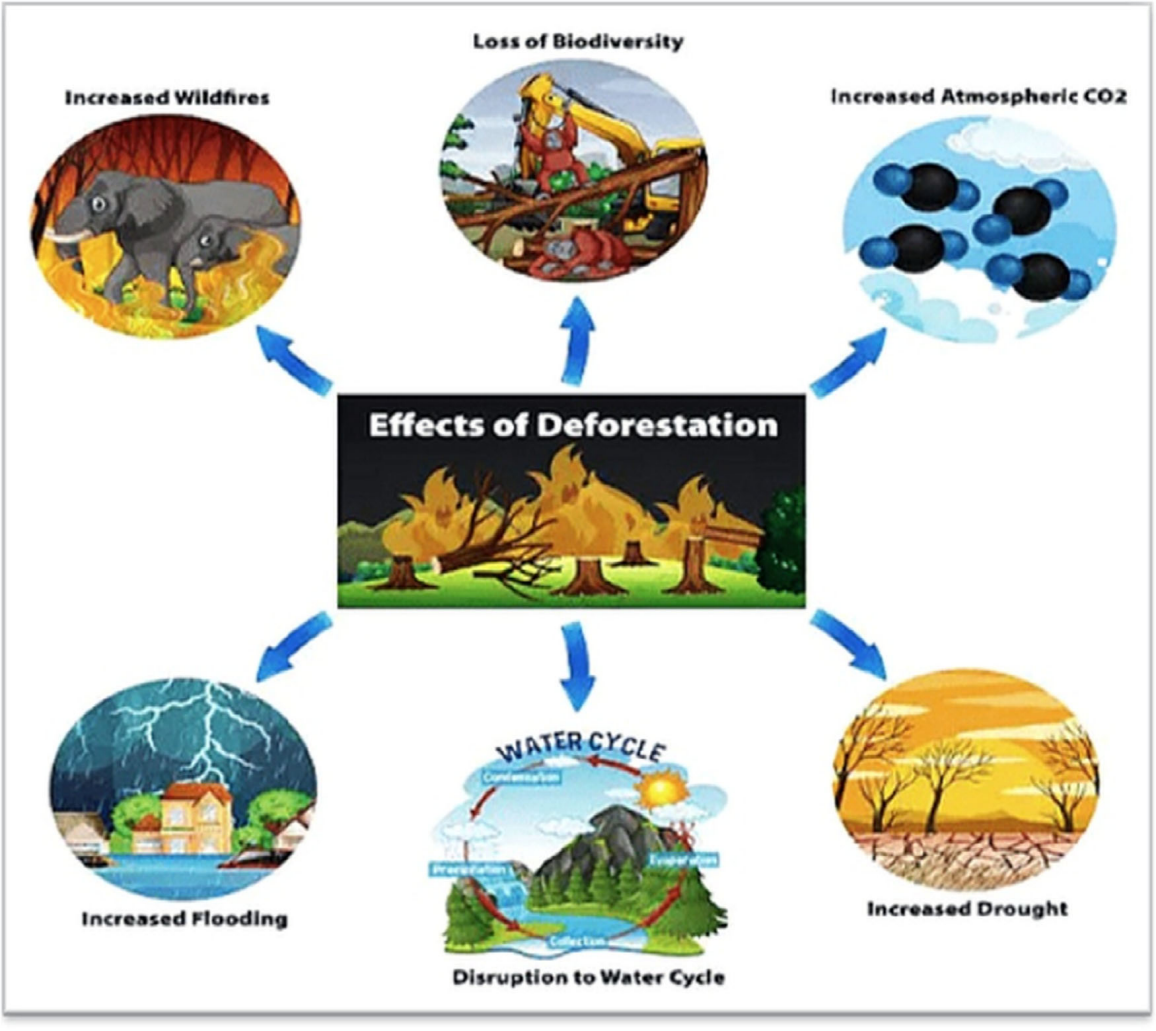
Environmental implications of deforestation in Ghana. Source: Patil Amruta collection on deforestation

Deforestation leads to significant environmental impacts, including biodiversity loss, climate change, water cycle disruption, global health issues, desertification, economic impact, soil erosion, and degradation, extending beyond national borders (Butler, 2019; Adolph et al. 2023). Consequently, the implications and consequences of these actions extend beyond national boundaries (Butler, 2019). Deforestation in Ghana significantly impacts the environment and social and economic livelihoods and contributes to the Earth’s climate system by acting as a carbon sink (Amoah et al. 2019; Brack 2019). Deforestation in Ghana has led to increased temperatures and changes in rainfall distribution, creating unpredictability in climatic conditions, as it interacts with global warming caused by greenhouse gas emissions (Cudjoe et al. 2021; Li et al. 2022). Figure 2 summarizes the environmental impacts of deforestation experienced in Ghana.

**Fig. 3:**
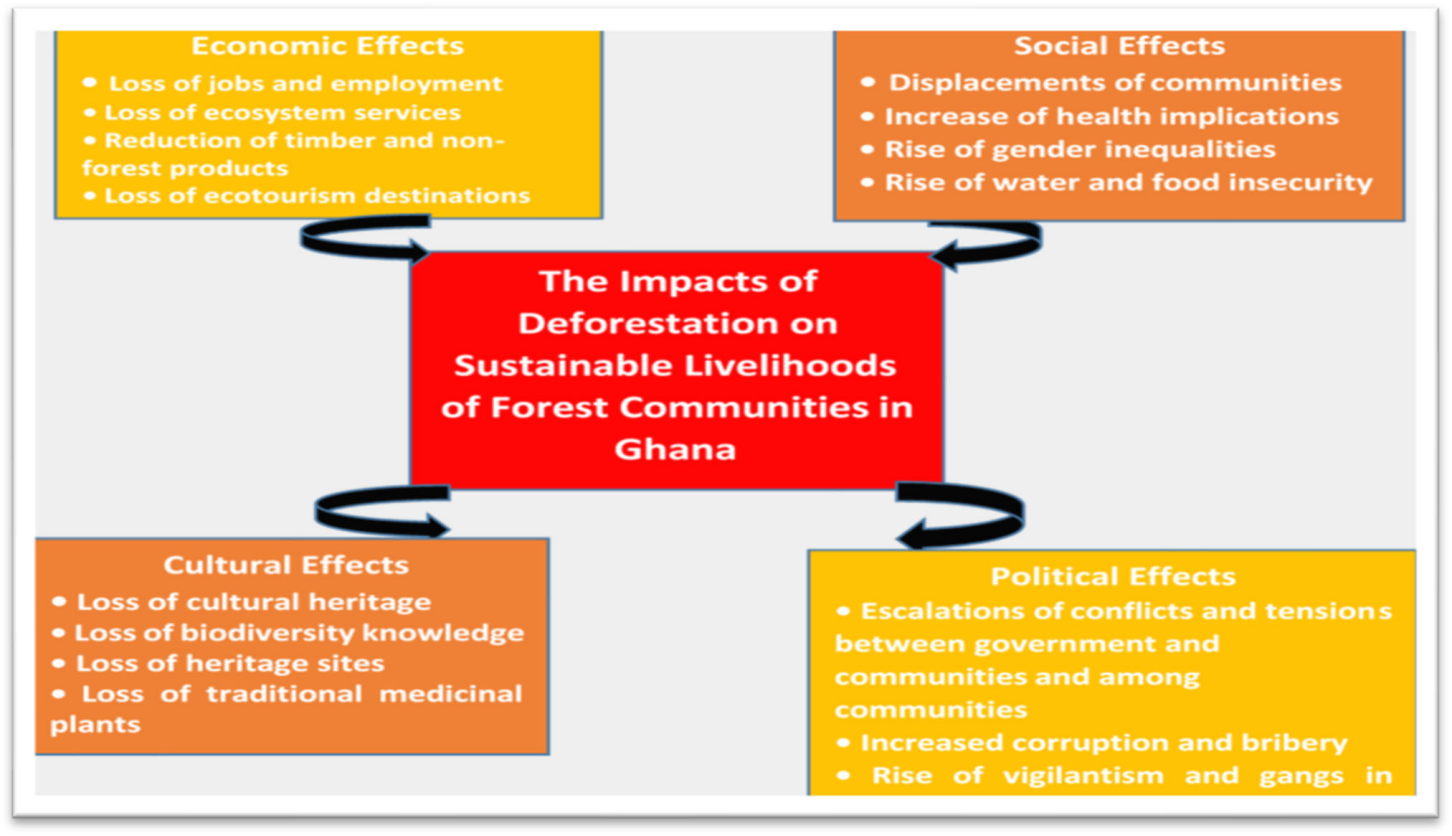
Implications of deforestation on livelihoods in Ghana. Source: Field survey 2023

Deforestation in Ghana has significantly reduced biodiversity over the past five decades, particularly in tropical rainforests, which are crucial habitats for millions of organisms, causing significant loss (Butler 2019; Jackson et al. 2020; Shivanna 2022). Over 60% of Ghana is covered by forests, housing 80% of all animal and plant species. Deforestation destroys these forests, affecting the genetic diversity of the species that inhabit them (Butler 2019; Baidoo et al. 2023). Deforestation and genetic diversity decline, impacting ecosystem health and species diversity, while trees regulate water flow, prevent soil erosion, and preserve water resources (Wiekenkamp et al. 2019; Muluneh, 2021). Deforestation has severely impacted the country’s land cover, exposing it to extensive wind and rain, thereby increasing soil vulnerability and proneness to erosion (Butler 2019). Deforestation significantly impacts social, economic, and political sustenance, especially for Indigenous and rural communities in Ghana, who rely on forests for livelihoods such as food, medicines, fuelwood, and jobs (Wale et al. 2022). Deforestation leads to displacement and migration of communities reliant on forests, causing cultural loss, loss of community cohesion, and reduced access to resources and land (Pearson et al. 2021). Furthermore, the loss of forests has resulted in a decrease in the availability of essential forest resources like timber and non-timber forest products, which are crucial for community livelihoods. Similarly, Fagariba et al. (2018) discovered that deforestation impacts livelihood activities in agriculture, mining, wood logging, and hunting, affecting employment, food security, income, economic growth, family cohesion, migrations, and cultural identity. Unsustainable forest management leads to negative impacts on livelihood activities (Pearson et al. 2021). Figure 3 summarizes the implications of climate change on sustainable livelihoods.

Many of the above-mentioned implications are exacerbated by poor policies and weak regulations and monitoring systems (Asamoah et al. 2023). To enhance sustainable livelihoods and reduce deforestation, it is crucial to implement intensive education, awareness creation, and effective forest management strategies (Lopez-Carr 2021).

## Materials and Methods

In this section, research design, sampling and sample population, data collection and techniques, and data analysis were explored.

### Study Area

**Fig. 4:**
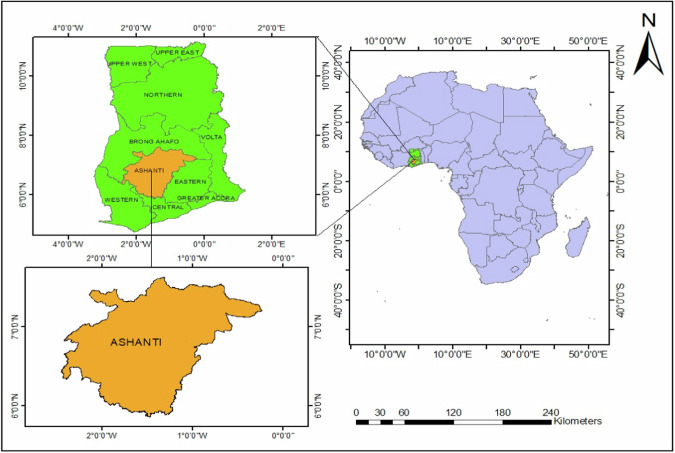
Map of Ashanti region in the southern part of Ghana

The study area is the Ashanti region in the Southern part of Ghana. Ashanti is located between 6°44’49.357”N and −1°31’15.105’E spanning 24,389 km^2^ (9417 sq mi) in Ghana (Fig. 4), with an approximately total population of 4,780,380 inhabit-ants (Khan-Andersen 2024). The region was chosen as a case study due to its geographical location, deforestation trends over the past three decades, and simultaneous socioeconomic conditions across the country. The climatic condition in the region is sub-tropical, with an average rainfall of between 1100–1800 mm, a temperature range of 21° to 32 °C, and higher humid conditions (Amponsah et al. (2002)). The Ashanti region, a hub for commercial crops like cocoa and palm trees, is characterized by a mix of cropland and tree cover, with the Atewa Range Forest Reserve and Tano-Offin Forest Reserve being the most dominant (Appiah and Guodaar 2022). The region’s social and economic dynamics, along with population growth, energy costs, poverty, and unemployment, significantly impact the use of natural resources, particularly forests, leading to exponential deforestation (Kyere-Boateng et al. 2021).

### Research Design

The study utilized a mixed-method approach, combining quantitative and qualitative methods, to gather data on the extent of deforestation in Ghana. In addition, the study conducted a comprehensive literature review on the impact of deforestation on climate change and the social and economic livelihoods of the population. The study utilized geospatial data analysis to examine the rates and trends of deforestation in the region between 2000 and 2020, using a quantitative technique (Afuye et al. 2022; Mpanyaro et al. 2024). The approach involves exploring the relationship between demographic patterns and the rate of deforestation in the study area. The study utilized a qualitative approach through interviews to evaluate the effects of deforestation on the social and economic livelihoods of the population and identify the causes and drivers of deforestation in the region (Enbakom et al. 2017; Ken et al. 2020). Additionally, the study extensively reviewed the existing policies and regulations in the region and country to evaluate their effectiveness and applicability in addressing deforestation issues.

#### Remote sensing data acquisition

Remote sensing data from Terra Moderate Resolution Imaging Spectroradiometer (MODIS) Normalized Difference Vegetation Index (NDVI) MOD13Q1 and 16-Day L3 Global 250 m imagery between 2000 and 2020. The Modis data was downloaded from the United States Geological Survey (USGS) Earth Explorer (https://earthexplorer.usgs.gov) for the analysis. The Ashanti boundary shapefile, sourced from Esri, was utilized to determine the scope of analysis on the area of interest (Zhu and Woodcock 2012; Wang et al. 2020). The satellite-derived vegetation index such as the NDVI for the respective years under review was clipped using the Ashanti boundary shapefile to narrow down the analysis to the coverage of the study. This was done with the clip option in the geoprocessing tool of ArcGIS. The MODIS NDVI data values range from −1999 to 10,000, and −2000 is the fill value. Value “−1999” is assigned to any VI computation between “−1998” and “−10,000”. To normalize the values, a scale factor of 0.0001 is applied to the pixels by multiplication (Zhu and Woodcock 2012). The NDVI resulting values were used for further analysis. For the study, the NDVI imageries were classified into four classes with values ranging from –0.1 to 0 grouped having no vegetation, 0–0.2 as sparse vegetation, 0.2–0.5 as moderate vegetation, and 0.6–1 as dense vegetation being the class containing the trees and forest. The different classes of the imageries were reclassified to unify the range of pixel values within each class (Zhu and Woodcock 2012), for further processing. The no vegetation class, sparse vegetation class, moderate vegetation, and dense vegetation were assigned the values −1, 1, 2, and 3 respectively.

#### Sampling techniques and study population

The study utilized probability in simple random sampling and non-probability in purposive sampling technique for data collection. The complexities of deforestation activities in Ghana and their impact on social and economic livelihoods necessitated the gathering of diverse perspectives and viewpoints. According to Campbell et al. (2020), purposive sampling is the intentional selection of informants based on their special knowledge and expertise and the willingness and ability to elucidate a specific theme, concept, or phenomenon. This sampling technique was used to select a total of 15 participants for interviews for the qualitative aspect of the study. Based on research questions, the researchers applied judgemental criteria to select and engage in online interviews with a total of seven respondents. The group included employees from the Ministry of Land and Natural Resources, five from the Forestry Commission of Ghana, and three Ashanti region community leaders. This study utilized simple random sampling to collect quantitative data through a survey, ensuring equal probability of each sample being selected (Marradi, 2022). The author asserts that the simple random approach aims to prevent any potential bias in the representation of the entire population. This technique was considered because it assisted the researchers in sampling a large pool of respondents for the survey. A total of 1000 respondents were selected from five communities, namely, Konongo, Bekwai, Obuasi, Agogo, and Abetifi, all in the Ashanti region. To achieve the proportional representation of the population based on socioeconomics, the Probability Proportional to Size sampling is computed in Eq. (1).

1 $${\rm{P}}=({\rm{C}}/{\rm{T}})* {\rm{S}}$$

where, s was employed to compute the sampling population in each community. In this formula, P is the proportion of the community in the sampled population, C is the community population, T is the total population, and S is the sample size. Using this Eq. (1) for the analysis, the latest statistical information was obtained from StatsGhana (Ghana Statistical Service GSS (2021)). The communities identified include *Konongo* ≈ *41,238; Bekwai* ≈ *7267; Obuasi* ≈ *175 043; Agogo* ≈ *28,271; and Mampong* ≈ *79,726. The computation of the: Konongo (41,238/331,545* *×* *1000* = *124); Bekwai (7267/331,545* *×* *1000* = *22); Obuasi (175,043/331,545* *×* *1000* = *528); Agogo (28,271/331,545* *×* *1000* = *86) and Mampong (79,726/331,545* *×* *1000* = *240)*. Nonetheless, the study used a systematic sampling method, selecting respondents aged 18 or older who had resided in the community for at least two years. The questionnaires were self-administered with the help of two field assistants who were conversant with the area.

## Data Analysis

**Fig. 5:**
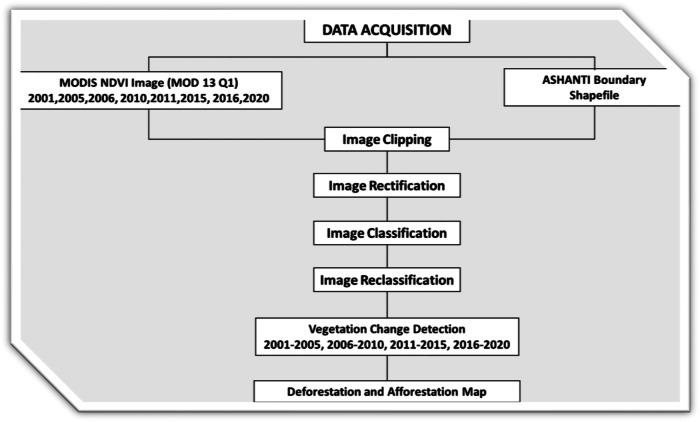
Workflow of the image classification methods

The study utilized remote sensing and GIS to analyze forest cover change in the Ashanti region, assessing the quality of the green vegetation condition index from 2001–2020. The Normalized Difference Vegetation Index was utilized to measure vegetation cover change and deforestation events in Ashanti, enabling a comprehensive mapping of vegetation cover status over time.

for the years 2000 to 2020. The NDVI themes used in the image classification method represent the most consistent temporal gaps within the region, as depicted in Fig. 5. The selection of pixels was made to capture a clear and significant cover change trajectory, considering the response time of landscape transformation to land cover changes (Shawul and Chakma 2019; Aspinall et al. 2021). The data was collected and analyzed between 2000 and 2020 using the intersect geoprocessing method for change detection (Bashir and Ahmad 2017). The method was employed to assess the pixel level at various changes in the landscape over time (Rajkumar 2022). The results were utilized for a change detection analysis of historical forest cover and the rate of vegetation state transitions (Fig. 5).

In addition, the study utilized descriptive statistics of SPSS Windows Version 21 to analyze questionnaire data, focusing on demographic characteristics, the socioeconomic status of households, and the impact of deforestation on livelihoods (Kiger, Varpio (2020)). The research team utilized SPSS software for data analysis, creating visual aids like frequency Tables and graphs and thematic analysis method for qualitative data from interviews and literature review. Thematic analysis is a method used to analyze qualitative data by identifying key messages or themes from interviewees, aiding researchers in sorting and grouping data for interpretation and discussion (Kiger, Varpio (2020)). The study utilized a Pearson correlation coefficient to establish strong connections between deforestation and sustainable livelihoods among the population in the region and country (Lahai et al. 2022; Nyirarwasa et al. 2024). The Pearson correlation coefficient is computed in Eq. (2).

2 $${{r}}=\frac{\sum_{i}({X}_{i-{\bar{X}}})({Y}_{i-{\bar{Y}}})}{\sqrt{\sum_{i}({{X}_{{i-}{\bar{X}}}})^{2}}-{\sum ({Y}_{{i-}{{\bar{Y}}}})^{2}}}$$

where, ${X}_{i}$ represent the amount of forest area lost due to deforestation over a specific period, $\bar{X}$ is the mean value of deforestation, ${Y}_{i}$ represent the values of sustainable livelihoods, such as the income or employment rate of the population in the region and $\bar{X}$ is the mean value of sustainable livelihoods with a value $r$ ranging from –1 to 1 respectively. To evaluate the impact of deforestation on the environment, and economic, cultural, and social livelihoods of the population at different time intervals, the Multiple Linear Regression (MLR) model was utilized for the study. The MLR has been widely used to evaluate multiple factors of deforestation affecting sustainable livelihood measures (Van Khuc et al. 2018; Soe and Yeo-Chang 2019). The MLR is calculated in Eq. (3).

3 $$Y={\beta }_{0}+{\beta }_{1}{x}_{1}+{\beta }_{2}{x}_{2}+{\beta }_{3}{x}_{3i}+\cdots +{\beta }_{n}{x}_{n}+{\varepsilon }_{i}$$

where, ${Y}$ is the dependent variable (i.e., deforestation), which is a function of the linear combination of the independent variables ${x}_{1,}{x}_{2}\ldots {x}_{n}$ (e.g., environmental, economic, social, and cultural implications) and ${\beta }_{0}$ is the intercept, while ${B}_{1,}{B}_{2}\ldots {B}_{n}$ are the coefficients representing the change in $Y$ for a one-unit change in each $x$ and ${\varepsilon }_{i}$ is the error term.

### Empirical Evidence and Analysis of Results

This analysis was carried out to analyze the causes and drivers of deforestation, its impact on social and economic livelihoods, and strategies for minimizing forest loss in the Ashanti region of Ghana (Asibey et al. 2020). The empirical evidence and analysis were computed by Eq. (4).

4 $$\sigma =\sqrt{\frac{\sum I{{X}}_{1}-\mu {I}^{2}\,}{N}}$$

where, σ is the standard deviation, x_1_, µ is the mean, and N is the total number of respondents.

### Demographic and Socioeconomic Status of Respondents

This paper explores socioeconomic and demographic factors to understand the drivers of deforestation in the Ashanti region of Ghana, using the above formula for computation. Table 1 shows a comprehensive overview of the demographic and socio-economic data of the respondents.

**Table 1:** Demographic and socioeconomic background of respondents

Variables	Frequency	Per. (%)	Mean (SD)	Mean per. (%)	Standard Deviation	Standard Error
Gender						
Female	300	30	250	12%	0.67	0.14
Male	700	70	250	28%	0.28	0.02
Age						
18–30 years	240	24	100	24%	0.17	0.12
31–40 years	340	34	100	24%	0.41	0.01
41–50 years	170	17	100	17%	0.18	0.08
51–60 years	144	14	100	14%	0.39	0.05
60^+^ years	106	11	100	11%	0.90	0.22
Educational levels						
No formal education	115	16	250	6%	0.93	0.08
Secondary level	540	54	250	22%	0.54	0.20
Bachelor degree	250	25	250	10%	0.65	0.45
Postgraduate	95	10	250	4%	0.98	1.12
Marital status						
Single	210	21	125	17%	0.19	0.01
Married	560	56	125	45%	0.55	0.03
Divorced	150	15	125	12%	0.67	0.29
Not disclosed	80	08	125	6%	1.02	0.16
Employment status						
Employed	214	24	125	19%	0.17	0.12
Self-employed	336	34	125	27%	0.26	0.02
Unemployed	420	42	125	24%	0.49	0.03
Seasonally employed	10	01	125	0.8%	1.25	0.56
Nature of employment						
Cash crop production	273	27	143	19%	0.296	0.13
Subsistent farming	260	26	143	18%	0.184	0.14
Mining sector	146	15	143	10%	0.187	0.23
Logging/pit sawyers	201	20	143	14%	0.185	0.13
Charcoal producers	90	09	143	06%	1.040	0.67
Forest rangers	20	02	143	01%	2.034	0.78
Others	10	01	143	01%	5.235	0.88

Source: Field survey 2023

Table 1 shows the survey of 1000 respondents, 700, equating to 70%, were males compared to 300 or 30% females. The majority, 340 (34%) of the population, were between the ages of 31 and 40 years; this was followed by the ages of 18–30, constituting 24%, and the majority, 54% of the respondents, finished secondary and bachelor’s degrees. Most of the respondents (56%) were married, 21% were single, and 15 were divorced. One of the most striking findings showed that 42% of the respondents were unemployed, followed by 34% self-employed and 24% employed. The outcome of the survey further shows that 27% of the respondents were involved in cash crop production such as cocoa and palm trees; this is immediately followed by subsistent farmers of 26%, logging pit sawyers came out next at 20% while mining both small-scale and large scale were mentioned by 15% of the total respondents.

#### Long-term inter-annual deforestation trends in the Ashanti region from 2000 to 2020

**Fig. 6:**
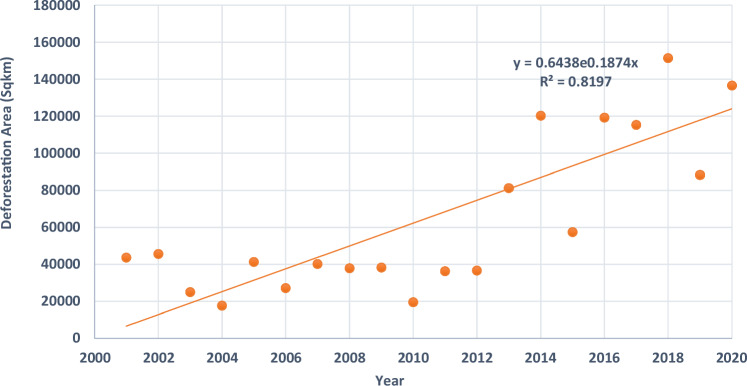
Long-term inter-annual deforestation trends in the Ashanti region from 2000 to 2020

Figure 6 shows the trends of deforestation in the Ashanti region of Ghana. The study used the MODIS vegetation indices time series product (MOD13Q1) from 2000 to 2020 to assess long-term inter-annual deforestation trends in Ghana’s forest areas since 2000. The results revealed an increasing rate of deforestation which became apparent in 2014 and 2020. The increasing trend can be attributed to the expansion of cities in the Ashanti region of Ghana. The trend line equation indicates an exponential increase in deforestation area over the years, with a value of 0.6438e^0.1874x^ square kilometers. The *R*² value of 0.8197 indicates a strong correlation between the years and deforestation areas, confirming the accuracy of the trend line in the exponential model (Fig. 6). The deforestation trend in the year 2018 peaked at around 16,000 (Sqkm) which might be linked to the large-scale clearing of forests and trees for commercial purposes including exports to global markets. Similarly, an increasing trend of deforestation was reported and linked to the establishment of new communities and infrastructural development that support the growing population and economic growth (Puplampu and Boafo 2021). These authors disclosed that between 2008 and 2021, urban built developments have expanded from 55% to 84% at why the expense of the natural environment, including green spaces, which have declined from 41% to 15% over the same period. Puplampu and Boafo (2021) argued that forest zones are the major target for infrastructure development. Moreover, the state’s policies promoting oil exploitation, logging concessions, and hydropower dam constructions have both intentionally and unintentionally led to significant deforestation in various regions of the country. Consequently, technological advancements, both modern and primitive, have significantly impacted deforestation, with inefficient logging technologies causing further damage to forests (Yilmaz and Koyuncu (2019)).

Of concern now is that forest crime and corruption have and continue to contribute to deforestation in Ghana (Cozma et al. 2023). The illegal logging and processing of forest raw materials across borders, facilitated by this phenomenon, has severe implications for deforestation in Ghana. In 2009, Ghana legalized informal small-scale logging under the European Union Forest Law Enforcement, Governance and Trade (FLEGT) and Voluntary Partnership Agreement, despite illegal forest activities being illegal in the country (Arts and Babili (2012)). The FLEGT and VPA programs have successfully increased consultation and participation of the private sector and civil society in forest governance, thereby enhancing forest sector transparency (Weisse et al. 2022). However, these strategies have not effectively promoted forest governance reforms and motivated sector agents to actively participate in forest conservation and tree cultivation (Weisse et al. 2022). Moreover, the forest management project alienated local communities from ownership, reducing them to passive subjects with limited rights and influence, leading to an increase in small-scale illegal logging (Kyere-Boateng et al. 2021). Hence, the Ashanti region of Ghana experienced a significant increase that contributed to the exponential trend in deforestation activities from 2014 to 2020, attributed to various factors, as depicted in Fig. 6.

#### Spatiotemporal analysis of vegetation cover and deforestation patterns in the Ashanti region

**Fig. 7:**
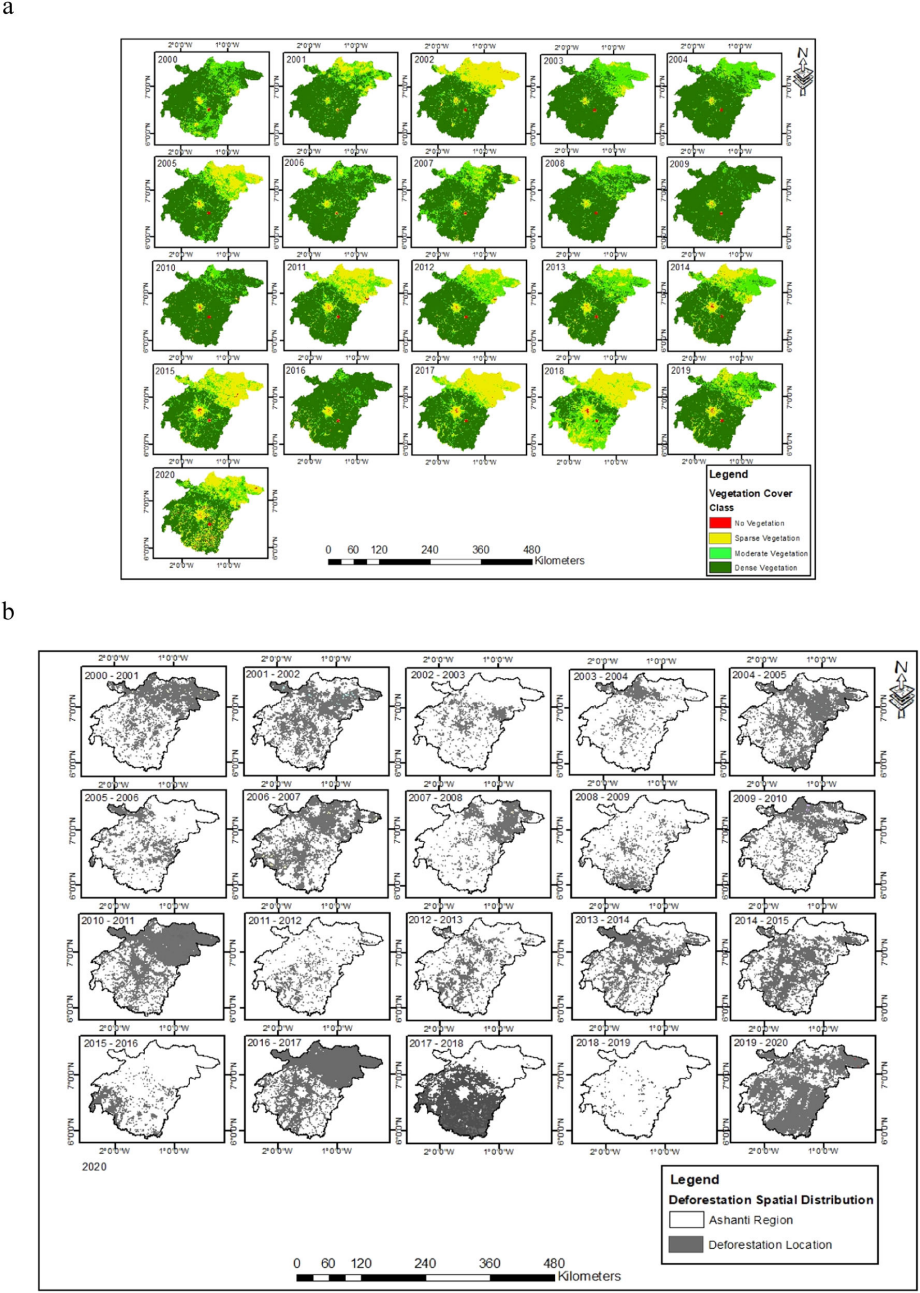
**a** Spatiotemporal analysis of vegetation cover, and (**b**) deforestation patterns in the Ashanti region from 2000 to 2020

Understanding the deforestation trends in the Ashanti region and Ghana forms one of the key objectives of the research. This was achieved using the statistics of forest loss and integrating remote sensing and Geographical Information Systems in the region between 2000 and 2020. Figure 7a, b shows the spatiotemporal analysis of vegetation cover and deforestation patterns in the Ashanti region. The vegetation cover status at the start of the millennium shows that the year 2000 experienced dense vegetation cover in the northeastern and southeastern parts (Fig. 7a). Most of the vegetation cover was sparsely dominated in the central while moderate vegetation was witnessed in the southern regions. It is worth noting that no vegetation was witnessed towards the southeastern parts of the study area. This connotes that the decline in tree and forest cover in this area might be due to the development of bare land, built-up, and/or urban areas. Nevertheless, dense vegetation cover dominated almost the entire region in 2000, with the most concentration in the southwestern parts of the Ashanti region (Fig. 7a). This might be linked to the improvements in vegetation cover change by the government that was fully undertaken in Ashanti because the issues with the land use/ land cover change and over-cultivation were still at an early stage during this period. It is possible that this development, which the province government implemented, had an impact on the vegetation cover, especially in 2000. The occurrence, therefore, aligns with Sustainable Development Goal SDG15 (Life on Land), which aims to protect, restore, and encourage sustainable land use and practices (Liu et al. 2019; Schillaci et al. 2023).

Additionally, deforestation trends in the Ashanti region between 2001 to 2005 show dense to moderate vegetation towards the central and southwestern parts (Fig. 7a). The region in the northeastern parts was dominated by sparse vegetation to no vegetation in the area. This may result from moderated land use and land cover change associated with human activities such as logging, mining, and urbanization being put under more stringent regulations and guidelines (Emenike et al. 2020; Afuye et al. 2024). From 2006 to 2010, the area witnessed moderate to dense vegetation towards larger areas in the central, southeastern, and western parts, while the northeastern part was dominated by less dense vegetation. Concurrent with the vegetation cover, the years 2002, 2005, 2011, 2015, 2017–2018, and 2020 witnessed moderate to sparse vegetation and sparse to no vegetation in the northcentral part of the region. This may be attributed to intense human disturbances that might have contributed to the observed vegetation cover dynamics (Afuye et al. 2021a). Additionally, the years 2011 to 2015 experienced a consistent trend and changes from moderate to sparse vegetation cover in the north and central parts. It is worth noting that 2018 witnessed moderate to sparse vegetation cover, and much of the changes were observed, while 2020 revealed dense to sparse vegetation in the southeastern parts of the Ashanti region. The observed built-up area became apparent in 2010, which may be due to the effects of arable farming, demographic pressure on land use change, and infrastructural development in the area (Koranteng and Zawila-Niedziecki (2015); Acheampong et al. 2019).

Figure 7b shows the spatiotemporal analysis of deforestation patterns in the Ashanti region between 2000 to 2020. Deforestation during 2000–2005 was primarily concentrated in northeastern areas with sparse vegetation cover, with some years showing more deforestation. From 2006–2010, deforestation patterns in northeastern areas with sparse vegetation cover expanded and intensified, affecting larger areas, indicating both expansion and intensification of deforestation activities. From 2011–2015, deforested areas became more widespread, with some regions experiencing higher concentrations. In previous decades, the dense to sparse vegetation was much noticeable in the central and northwestern areas, particularly when the land use changes were just in the early phase. From 2016–2020, deforestation in the region increased significantly, with a high peak in 2020, primarily in the southeastern parts, indicating a pervasive spatial distribution. The southeastern part was dominated by sparse to no vegetation as opposed to the previous years under investigation. The increasing gray areas indicate the expansion of deforested lands, underscoring the urgency of addressing deforestation and implementing sustainable land management practices (Fig. 7b). Consequently, anthropogenic climate change exerts a significant influence on tree cover and forest cover change, which might have transformed the ecological landscape (Afuye et al. 2021b; Wang et al. 2020). This development might have influenced the transformation of forest to other land, which could have impacted the soil structure associated with tree loss and forest cover change that occurred from dense vegetation to sparse in the region (Fig. 7b). A comprehensive strategy for managing forest vegetation and mitigating drivers of deforestation under the concepts of ecosystem-based adaptation is needed to effectively address the challenges posed by environmental hazards in the country and any region (Busayo et al. 2022). The information-based model for sustainable ecological conservation and restoration policy must thus be included in comprehensive strategies to manage a range of activities that may be caused by both natural and human activities (Afuye et al. 2021a).

#### Proximate causes of deforestation

**Fig. 8:**
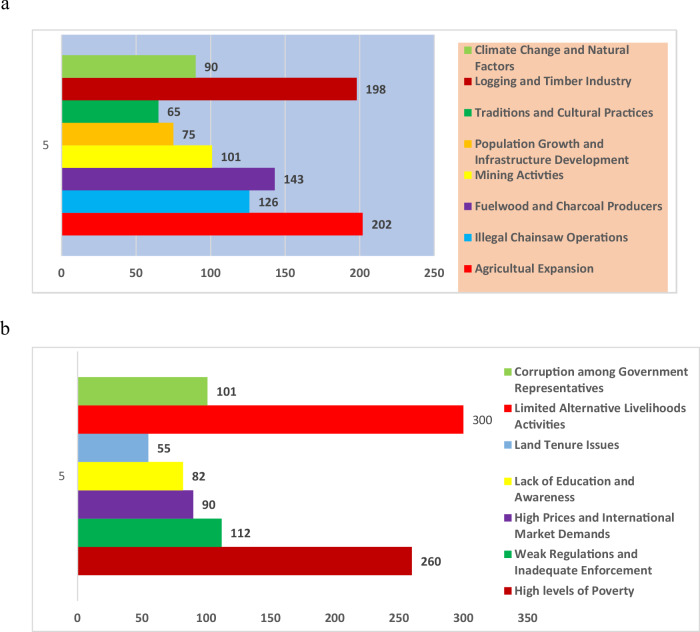
**a** Drivers of deforestation in the Ashanti region. **b** The underlying drivers of deforestation. Source: Field survey by Researchers

This study aimed to identify the primary factors contributing to deforestation in the region. Respondents were asked to identify the main causes of deforestation in their communities. The responses of the respondents are depicted in Fig. 8a.

Cultural activities that contribute to deforestation in Ghana include shifting cultivation, fuelwood and charcoal production, weakening sacred grove protection, rituals, traditional land tenure systems, hunting, gathering, and unsustainable plant harvesting for traditional medicine (Boafo 2013; Fagariba et al. 2018; Butler, 2019; Asamoah et al. 2023). However, the majority of the respondents, 20%, mentioned agricultural expansion and logging and timber industry as the main and immediate causes of deforestation in their communities (Fig. 8a). This was followed by fuelwood and charcoal producers at 14% and illegal chainsaw operations, and mining activities were mentioned at 13% and 10% respectively. This statistical breakdown was reinvigorated by a community leader who was engaged in an interview. This interviewee posited that:

“the enticements and the drive by government to farmers to go into large-scale production of cocoa, palm, and rubber have contributed to the clearing of large portions of forests to make space for the cultivation of these products. This interviewee further said it is cheaper to buy forest land and turn it into farmland in the Ashanti region than to invest in existing farmland to improve productivity and longevity”.

Another interviewee from the Ministry of Land and Natural Resources shared a similar perspective. This interviewee stated in an interview that:

“large scale and illegal logging activities for sawmill industries and other purposes are the primary contributor to deforestation in the country and Ashanti region in particular. According to this interviewee, logging operations employ clear-cutting; in some instances, entire sections of the forest are completely cleared off. These practices have resulted in massive deforestation in the region”.

Apart from the proximate causes, the study probed further into the underlying drivers of deforestation in the region. Respondents were asked to identify the root causes of forest loss in their communities. Figure 8b displays the answers of the respondents.

Most of the respondents (30%), attribute limited and no alternative to livelihood opportunities in their communities as the key driver to deforestation (Fig. 8b). This is followed by pervasive poverty; this factor was mentioned by 26% of the respondents. Several respondents, 11% and 10% respectively, associated weak regulations, inadequate enforcement, and corruption among government officials as underlying drivers of deforestation in communities in the country and the region. Some interviewees buttressed these statistical outcomes. An employee from the Forestry Commission in Ghana said this during an interview:

“most of the rural or local communities in Ghana and Ashanti region lack any other income source besides clearing the forest or logging by legal or illicit means. This dependency on forest-related sustenance has contributed substantially to unsustainable deforestation”.

Another employee from the Regional Ministry of Land and Natural Resources expressed similar sentiments:

“the majority of the population in rural communities in the country are poor and have no alternative livelihoods; they rely mainly on forest resources for sustenance such as charcoal production, cutting of trees for fuelwood, hunting and gathering, illegal mining activities, illicit logging and subsistence in the form of slashing and burning. These collective activities contribute to deforestation as forests are overly exploited”.

An Environmentalist and an Advocator based in one of the communities expressed this during an interview:

“There is a lack of transparency in the Ghana forestry sector and this is contributing significantly to deforestation in all the regions in the country. This interviewee stressed that the opaque practices in the logging permits, land concessions, and timber harvesting have contributed to illegal and unsustainable deforestation. The lack of transparency has hindered any effort to enforce regulations and sustainable forest management in the country”.

A common perspective cutting across most of the respondents suggests that favorable markets for forest products from both domestic and global, weak regulations on forestry, lack of awareness, and complications of the land tenure system have collectively contributed to the escalation of deforestation in the region and Ghana as shown in Fig. 8b.

#### Implication of deforestation on the environment, social and economic livelihoods

This study evaluated the implications of deforestation on the livelihoods of the population. Respondents were asked to link the implications against a particular variable using a set of variables, with responses presented in Table 2. The following equation was used to compile the ranking: [0 < x < 20 equal to (No implications); 21 > x < 100 (Less implications); 101 > x < 500 (Major implications); 501</ = 1000 (Very severe implications)]. Table 2 depicts the impacts of deforestation on the environment and livelihoods

**Table 2:** Implications of deforestation on sustainable livelihoods

Nature of constraints										
	No Implications	Per. %	Less Implications	Per. %	Major Implications	Per. %	Very Severe Implications	Per. %	Total no. of Respondents	Total Per. %
Environmental Implications										
Loss of Biodiversity	10	1%	60	6%	380	38%	550	55%	1000	100%
Expansion of Soil Erosion	17	2%	50	5%	400	40%	533	53%	1000	100%
Modification of Climate Change	25	3%	88	9%	654	65%	233	23%	1000	100%
Impact on Water and Air Quality	15	3%	63	49%	412	30%	510	51%	1000	100%
Economic Implications										
Loss of Jobs and Employment Opportunities	16	2%	34	3%	327	32%	623	62%	1000	100%
Reduction in Eco-Tourism Destinations	23	2%	67	8%	399	40%	511	51%	1000	100%
Reductions of Agricultural and Forest Produce	9	0.9%	25	3%	517	52%	449	45%	1000	100%
Reductions Revenue from Timber and Non-Timber Products	11	1%	67	7%	412	41%	510	51%	1000	100%
Social and Cultural Implications										
Increases Displacement and Migration	76	8%	150	15%	320	32%	454	45%	1000	100%
Loss of Cultural and Traditional Knowledge	88	8%	286	29%	380	38%	246	24%	1000	100%
Increases Health, Water, and Food Insecurity	19	5%	67	8%	226	23%	688	69%	1000	100%
Escalation of Conflict and Gangstars	206	20%	440	44%	186	19%	168	17%	1000	100%

Source: Field survey 2023

Most of the respondents classified the twelve implications as either major or very severe implications on sustainable livelihoods (Table 2). For instance, 62% of the respondents are of the view that deforestation has contributed to the loss of jobs and unemployment, escalating poverty in the region and the country. Similarly, 69% mentioned that their health, water, and food security have been compromised due to high levels of deforestation. Interviews with some respondents buttressed the statistical breakdown. An interview with a community leader stated that:

“Our livelihoods depend on timber, non-forest products, ecotourism, and the hospitality industry. The decline in forest in our communities reduced attractions, limits the supply of timber which adversely affects employment for loggers, sawmill workers and other industry”.

The underlying views emanating from most interviewees suggest that deforestation in the region is multifaceted. Deforestation disrupts the water cycle, which compromises water quality and quantity, leading to the outbreak of diseases; it also disrupts the ecosystem and biodiversity, reducing agricultural production, food insecurity, environmental concern, and human well-being.

#### Mitigation strategies for deforestation in the Ashanti region and Ghana

**Fig. 9:**
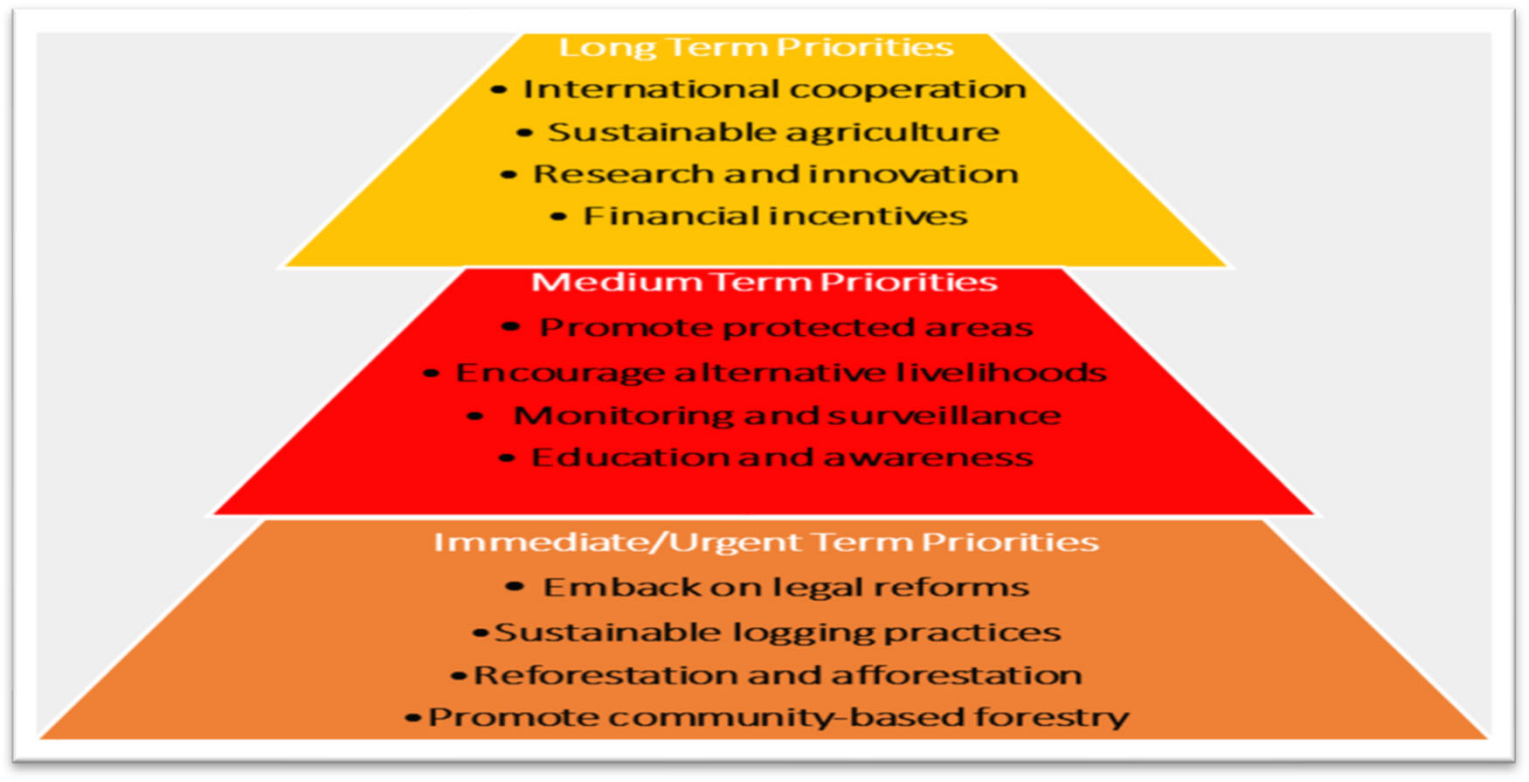
Categorization of mitigation strategies for deforestation. Sources: Field survey 2023

Considering the negative implications of deforestation on the population’s livelihoods, this study explored strategies for mitigating the adverse effects of deforestation in the Ashanti region. Respondents were asked to pick their preferred strategies for addressing deforestation challenges from a set of variables (Fig. 9). Using a “Likert scale”, we calculated and classified respondents’ answers under the following variables: “Priority, Important Priority, and Very Important Priority”. Under this formula, we use a normal and ordering approach of (< less than; = equal to; > greater than; ≤ less or equal to ≠ not equal to; ≥ greater or equal to and x to equate numbers within a range) to calculate the answers provider. In this equation, responses were compiled as: [0 ≤ x ≤ 100 equal to “Long Term Priority”; 101 ≤ x ≤ 500 equate to “Medium Term Priority” and 501 ≤ x ≤ 1000 represent “Immediate/Urgent Priority”]. The responses are depicted in Fig. 9.

Respondents classified legal reforms, sustainable logging reforestation, and afforestation, as well as community-based forestry, as immediate or urgent priorities that need to be enforced if the region and the country are to address the challenges of deforestation (Fig. 9). These were followed by promoting protected areas, encouraging alternative livelihoods, monitoring, and surveillance, and promoting education and awareness campaigns as medium-term priorities required in solving deforestation challenges. Other respondents grouped international cooperation, sustainable agriculture, research and innovation, and financial incentives as long-term objectives to be followed to solve deforestation problems in the country. These views were validated during interviews with some of the respondents. For instance, an interview with the director of the Ministry of Lands and Natural Resources posited that:

“legal reforms and strengthening existing laws such as land tenure rights, regulating logging and mining, encouraging community-based forest management, conducting effective environmental impact assessment. This includes improving forest monitoring with technology, harsh penalties for offenders and corrupt officials, and well collaborating with neighbouring and international agreements will go a long way to minimize deforestation activities in the region and the country”.

During an interview, an employee expressed a similar sentiment at the Forestry Commission of Ghana. This interviewee stated that:

“addressing deforestation activities in Ghana will require a holistic approach that embraces restructurings legal and regulations on forest management, responsible logging, community engagement, forest certification, corporate accountability, global cooperation as well as sustainable agricultural practices”.

A conservationist and Forester in the Ministry of Lands and Forestry said this in an interview:

“to slow the effect of climate change, conserve biodiversity, and impact the sustainable development goals, replanting and reforestation are critical. He stated that restored forests store carbon within the forest’s soil, shrubs, and trees. Mixed forests, for instance, are especially effective at carbon storage, as different species with complementary traits increase overall carbon storage”.

A common and underlying feedback emanating from the interviewees suggests that proactive and integrated strategies of robust legal regulations, active community involvement and ownership of forests, political will, and active educational and awareness campaigns will reverse the country’s deforestation trend.

### Discussion

#### Drivers and deforestation trends in Ashanti, Ghana

This study provides a comprehensive analysis of the drivers and trends of deforestation and its impact on Ghana’s environment and social and economic livelihoods. The findings of this study confirmed the views of many scholars, researchers, and commentators. From 2000 to 2020, the Ashanti region experienced relatively stable deforestation trends, with minor fluctuations, indicating a stable environment. From 2005, there was a noticeable increase, while between 2010 and 2015, there was variability with both increases and decreases. Post-2015, deforestation experienced a significant increase, peaking around 2018 and maintaining high levels through 2020. The Ashanti region is experiencing a rapid rise in deforestation, posing significant environmental challenges due to the pressure on forest resources and potential ecological degradation (Figs. 6 and 7a, b). The results demonstrate a clear and concerning trend of increasing deforestation in the region, with an exponential growth pattern that underscores the urgency for sustainable forest management practices. In addition, the current trend emphasizes the need for effective forest management and conservation policies to combat deforestation, such as Koranteng, Zawila-Niedziecki (2015), Welsink (2020), Wimberly et al. (2022), and Afele et al. 2022 that deforestation trends in the Ashanti region have varied since the 1950s, however, a significant forest loss has occurred between 2000 and 2022. The geospatial analysis of the region suggests that since 2001, the region has experienced fluctuating trends in deforestation at an astounding yearly rate of forest loss, which has increased over the study period. Similar findings are expressed by Wimberley et al. (2022), who noted that from 1990 to 2000, the rate of forest cover loss in the Ashanti region was 3%; between 2001 to 2010, the rate was 2.6% and 1.5% and 2.5% between 2010 and 2020. This paper identified two main drivers as the main anthropogenic causes of deforestation. These include proximate or immediate causes and underlying factors. Agriculture expansion, logging, timber extraction, fuelwood and charcoal production, illegal chainsaw operations, infrastructure development, and traditional and cultural practices are the region’s proximate or immediate causes of deforestation activities. The case study findings by Andoh and Lee (2018) support the claim that deforestation in Ghana, particularly in the Ashanti region, is primarily caused by cash crop expansion, unsustainable logging, illegal mining, fuelwood burning, and infrastructure development. The outcomes of the study suggest that the underlying drivers play a significant role in sustaining the proximate causes (Fig. 8a, b).

Furthermore, the study reveals that social, economic, and political factors such as poverty, weak regulations, corruption, land tenure systems, favorable international market conditions, and lack of education contribute to logging and deforestation. A similar outcome by Kyere-Boateng et al. (2021) discovered that while direct causes like agricultural expansion, logging, and mining are visible, underlying drivers like economic pressures, cultural circumstances, and political factors indirectly contribute to the expansion of deforestation in the region. Kissinger et al. (2012) argued that socioeconomic factors, bureaucratic licensing regimes, weak governance, political failures, corrupt officials, population growth, and global market forces all contribute to deforestation, leading to forest loss and environmental challenges. The findings from our engagements revealed a lack of focus on the underlying causes of deforestation, with many strategies focusing on addressing deforestation through stricter regulations or tree planting, without addressing poverty and lack of alternative livelihood strategies. Our engagements reveal that communities are largely excluded from forest management activities, despite conservation regulations and legislation recognizing communities and chiefs as forest custodians. The only way to protect and reduce deforestation activities in Ghana is by empowering stakeholders and ensuring their benefits from the forest and its products.

#### Implications of deforestation on sustainable livelihoods

Accordingly, deforestation has significant adverse impacts on the environment and sustainable livelihoods. The Pearson correlation coefficient was used to establish strong correlations between deforestation and sustainable livelihoods of the population in the region and country. The study reveals a significant negative correlation between deforestation and sustainable livelihoods, indicating that an increase in deforestation leads to a decrease in sustainable livelihoods (Table 2). Findings from Table 2 showed negative environmental impacts of deforestation, including loss of biodiversity, soil erosion, climate change, and reduced water and air quality. The study by Peprah et al. (2022) and Nyako et al. (2023) supports the notion that deforestation in Ashanti and Ghana results in significant environmental issues like biodiversity loss, habitat destruction, and climate change, thus affecting sustainable livelihoods. Studies uncovered negative impacts of deforestation that disrupts water cycles, reducing availability and quality, affecting air quality, and causing frequent vulnerabilities like flooding, droughts, and bushfires in urban areas further supporting our findings in Ghana’s Ashanti region (Acheampong et al. 2019; Amoah and Korle 2020; Sasu, 2022; Nyako et al. 2023; Asamoah et al. 2023). The findings of this study concurred with many commentators and researchers, such as Fagariba et al. (2018), Acheampong et al. (2019), and Siregar et al. (2023), that deforestation has far-reaching implications on economic livelihoods in the Ashanti region. The underlying findings in Tables 1 and 2 indicate that forest loss has contributed to the loss of jobs and employment opportunities, reduction in ecotourism destinations, reduction in agricultural production, and decrease in revenue for timber and non-forest products. The key findings in Table 2 reveal that the Ashanti region and Ghana’s economy heavily rely on agriculture, timber, and forest products, highlighting the direct impact of forest loss on employment, income, and livelihood activities. Furthermore, the extinction of forests in the region, due to their biodiversity, has reduced tourist interest, negatively impacting the hospitality and tourism sector, leading to reduced employment opportunities and business revenue. The findings of Yahaya et al. (2022) and Wale et al. 2022 report that deforestation in the region has led to significant job losses in the hospitality, travel, and service industries in local economies. Their study supports our findings on the significant social impacts of deforestation, including displacement, health risks, water and food security threats, cultural loss, and escalating conflicts in forest communities. Deforestation has led to the loss of traditional livelihoods, forced migration, health complications, and conflicts, particularly among youth, escalating vigilantism, and gangs, and threatening water and food security (Boafo 2013; Amoah and Korle 2020; Mulhneh 2021; Asamoah et al. 2023; Baffour-Ata et al. (2021)). The underlying findings of this study suggest that the environmental, economic, and social implications have collectively impacted the stability and well-being of communities in the region and the country as a whole.

#### Intervention strategies in mitigating deforestation

This study explored strategies required to address deforestation challenges in the region and the country. The findings recognized that mitigating the problems of deforestation will require the amalgamation of immediate or short-term to long-term strategies, as illustrated in Fig. 8a, b. The findings from Fig. 8 and studies by Knoke et al. (2022) suggest that the region should implement legal reforms, sustainable logging practices, reforestation, afforestation, and community-based forestry as immediate or short-term strategies to combat deforestation. Our views and observations suggest that current legislation and policies governing forest management in the country are characterized by neo-liberal, government-centered approaches, disregarding the interests of farmers, landowners, communities, traditional authorities, and other stakeholders in forest governance and administration. Furthermore, The legislation and policies lack clear direction on sustainable logging, reforestation, and afforestation programs required to maintain forest resources. To combat deforestation, policy and legal reforms should enhance forest governance, promote sustainable logging, ban illegal activities, protect biodiversity, and implement community-based management. These reforms will enhance law enforcement and biodiversity protection. These findings align with den Besten et al. (2019) framework for addressing deforestation and forest degradation under the Reducing Emissions from Deforestation and Forest Degradation (REDD+) in developing countries including Ghana, stating that no single stakeholder can effectively eradicate deforestation activities in the country (Fig. 8a, b and Table 2). Therefore, effective surveillance and monitoring require collaboration between government agencies, the private sector, especially sawmilling companies, and local communities.

## Conclusion and Recommendations

The study reveals that deforestation in Ghana’s Ashanti region is influenced by social, economic, political, and cultural factors, affecting the livelihoods of vulnerable communities and evaluating the impact of climate change. Additionally, the study analyzed vegetation cover and deforestation patterns from 2000 to 2020 using qualitative and space-based data. At the start of the millennium, the year 2000 experienced dense to moderate vegetation cover, while subsequent years witnessed a consistent decline in tree and forest cover. The study reveals a consistent decline in tree and forest cover quality from 2000 to 2013, exacerbated by landscape transformation response time in 2014, leading to a drastic decrease in vegetation quality. The study found a significant correlation between years and deforestation areas, particularly in 2018 and 2020, indicating an exponential increase with severe implications for sustainable livelihoods. Overall, between 2000 and 2020, the forest transformation showed a significant decrease in the quality of green vegetation conditions, from moderate to sparse. The findings of this study revealed a significant negative correlation between deforestation and sustainable livelihoods, indicating that an increase in deforestation leads to a decrease in sustainable livelihoods.

In light of the current trend of deforestation, poses significant environmental challenges due to the increasing pressure on forest resources and potential ecological degradation. Therefore, the exponential growth pattern underscores the need for sustainable forest management practices and effective conservation policies to combat deforestation. However, the Ghanaian government and traditional authorities have implemented various strategies and regulations to combat deforestation and anthropogenic climate change. Contrarily, the trends of forest loss are rather on the ascendency, particularly in the Ashanti region, indicating a growing environmental concern. The country’s forest management faces challenges such as siloed practices, community-driven strategies, corruption, poverty, and lack of alternative resources, exacerbated by environmental issues. The challenges at hand necessitate a holistic, integrated approach that includes addressing poverty, sustainable land use, and forest management. Promoting the use of trees like cocoa, coffee, and palm trees in farming can mitigate forest stress, maintain crop yields, and sustain the environment. The findings provide evidence-based and all-inclusive approaches to encourage marginalized groups to participate in policy and strategy co-production and co-creation. The outcome of this study is geared towards creating transformative and sustainable communities while ensuring efficient response and recovery capacities for deforested lands. Strengthening partnerships can be achieved by implementing innovative policies, upholding existing laws, and improving forest governance on both local and national levels. The goal is to improve forest conservation awareness and education, utilize technological advancements for efficient resource management, and meet population demands for social, economic, and livelihoods. Effective networked governance, including international organizations, the corporate sector, and local communities, is crucial for proactive actions and promoting sustainable agriculture.
